# Soft tissue manipulation enhances recovery of muscle mass in a disuse model of sarcopenia

**DOI:** 10.1515/jom-2024-0247

**Published:** 2025-03-13

**Authors:** Basil Mustaklem, Mary Terry Loghmani, Abigail K. Waterfill, Mackenzie Caron, Daren A. Glore, Nathaniel R. Meyer, Luke D. Shelton, Elicza A. Day, Carmela Marciano, Addison Gepfert, Connor C. Wakefield, Hailey Brown, Sierra Street, Madeline M. Sasse, Jacob Snyder, Taylor Hiland, Julia M. Hum, David C. Eland, Tien-Min Gabe Chu, Jonathan W. Lowery

**Affiliations:** Wood College of Osteopathic Medicine, Marian University, Indianapolis, IN, USA; and Bone & Muscle Research Group, Marian University, Indianapolis, IN, USA; Department of Physical Therapy, School of Health and Human Sciences, Indiana University, Indianapolis, IN, USA; and Indiana Center for Musculoskeletal Health, Indiana University School of Medicine, Indianapolis, IN, USA; Department of Physical Therapy, School of Health and Human Sciences, Indiana University, Indianapolis, IN, USA; Department of Physical Therapy, School of Health and Human Sciences, Indiana University, Indianapolis, IN, USA; Department of Physical Therapy, School of Health and Human Sciences, Indiana University, Indianapolis, IN, USA; Department of Physical Therapy, School of Health and Human Sciences, Indiana University, Indianapolis, IN, USA; Department of Physical Therapy, School of Health and Human Sciences, Indiana University, Indianapolis, IN, USA; Wood College of Osteopathic Medicine, Marian University, Indianapolis, IN, USA; and Bone & Muscle Research Group, Marian University, Indianapolis, IN, USA; Wood College of Osteopathic Medicine, Marian University, Indianapolis, IN, USA; and Bone & Muscle Research Group, Marian University, Indianapolis, IN, USA; Wood College of Osteopathic Medicine, Marian University, Indianapolis, IN, USA; and Bone & Muscle Research Group, Marian University, Indianapolis, IN, USA; Wood College of Osteopathic Medicine, Marian University, Indianapolis, IN, USA; and Bone & Muscle Research Group, Marian University, Indianapolis, IN, USA; Wood College of Osteopathic Medicine, Marian University, Indianapolis, IN, USA; and Bone & Muscle Research Group, Marian University, Indianapolis, IN, USA; Wood College of Osteopathic Medicine, Marian University, Indianapolis, IN, USA; and Bone & Muscle Research Group, Marian University, Indianapolis, IN, USA; Wood College of Osteopathic Medicine, Marian University, Indianapolis, IN, USA; and Bone & Muscle Research Group, Marian University, Indianapolis, IN, USA; Wood College of Osteopathic Medicine, Marian University, Indianapolis, IN, USA; and Bone & Muscle Research Group, Marian University, Indianapolis, IN, USA; Wood College of Osteopathic Medicine, Marian University, Indianapolis, IN, USA; and Bone & Muscle Research Group, Marian University, Indianapolis, IN, USA; Wood College of Osteopathic Medicine, Marian University, Indianapolis, IN, USA; Bone & Muscle Research Group, Marian University, Indianapolis, IN, USA; Indiana Center for Musculoskeletal Health, Indiana University School of Medicine, Indianapolis, IN, USA; and Indiana Biosciences Research Institute, Indianapolis, IN, USA; Wood College of Osteopathic Medicine, Marian University, Indianapolis, IN, USA; Department of Biomedical Sciences and Comprehensive Care, Indiana University School of Dentistry, Indianapolis, IN, USA; Indiana University School of Dentistry, Indianapolis, IN, USA; and Indiana Center for Musculoskeletal Health, Indiana University School of Medicine, Indianapolis, IN, USA; Wood College of Osteopathic Medicine, Marian University, 3200 Cold Spring Road, Indianapolis, IN, 46222, USA

**Keywords:** inflammation, manual therapy, muscle atrophy, rehabilitation, sarcopenia, soft tissue

## Abstract

**Context:**

Sarcopenia is a disease characterized by low muscle mass and function that places individuals at greater risk of disability, loss of independence, and death. Current therapies include addressing underlying performance issues, resistance training, and/or nutritional strategies. However, these approaches have significant limitations, and chronic inflammation associated with sarcopenia may blunt the anabolic response to exercise and nutrition. This presents an unmet need for treatment strategies that promote gains in muscle function. One such possibility is soft tissue manipulation (STM), which is a noninvasive, nonpharmacological mechanotherapy employed by osteopathic physicians, physiotherapists, and massage therapists, wherein soft tissues are subjected to mechanical forces delivered by hand or by an instrument. However, the molecular effects of STM in sarcopenia remain largely unknown.

**Objectives:**

In the present study, we utilized a rat model of sarcopenia due to disuse atrophy and examined the effects of STM on recovery of muscle mass and regulation of pro-/anti-inflammatory cytokines.

**Methods:**

Ten-week-old male Brown Norway rats were subjected to 2-week hindlimb suspension (HLS) and then allowed to re-ambulate for 8 days with or without instrument-assisted soft tissue manipulation (IASTM) applied to the right hindlimb. Muscle weights were determined for treated and nontreated hindlimbs, and membrane-based cytokine arrays were performed on treated tissue and serum.

**Results:**

Following suspension, IASTM enhanced the effectiveness of re-ambulation (Re-A) on muscle mass recovery in both treated and contralateral limbs. This was associated with changes in numerous cytokines in treated skeletal muscle and sera. Several factors we observe to be regulated were also shown to be regulated by STM in other studies, including ciliary neurotrophic factor (CNTF), IL-1β, IL-2, IL-3, IL-13, ICAM-1, and tumor necrosis factor alpha (TNF-α), whereas others are reported for the first time.

**Conclusions:**

Our study adds further support for the role of manual therapy in musculoskeletal health and details molecular-level effects in both target tissue and circulation. STM may hold promise for recovering muscle mass and function related in conditions of atrophy such as age-related sarcopenia.

Sarcopenia is a condition characterized by the age-related loss of muscle mass and function that diminishes physical capabilities and increases the risk of falls, fractures, and overall morbidity [1]. The prevalence of sarcopenia is estimated to be approximately 10 % in community-dwelling adults and three to five times higher among nursing home residents [2]. This condition places a substantial financial burden on the US healthcare system, with a total annual cost of hospitalizations related to sarcopenia of more than $40 million [3]. Despite significant research efforts, there are no approved pharmacological treatments for sarcopenia, and the current therapies primarily focus on lifestyle interventions, including resistance exercise and nutritional strategies [4]. However, these approaches have several limitations including adherence and accessibility, uncertainty on which type of intervention is most effective, and variability in effectiveness among individuals – particularly among patients with advanced sarcopenia [5–8]. Additionally, a growing body of evidence suggests that chronic inflammation may blunt the anabolic response to exercise and nutrition [9, 10].

Inflammation plays a crucial role in the development and progression of sarcopenia, and chronic low-grade inflammation is commonly observed in elderly individuals [11]. Such “inflammaging” is characterized by elevated levels of pro-inflammatory cytokines, which contribute to muscle catabolism by promoting protein degradation pathways and inhibiting muscle protein synthesis. Additionally, inflammation can impair the regenerative capacity of muscle stem cells, further exacerbating muscle loss [12]. The persistent inflammatory state not only accelerates muscle degradation but also interferes with the anabolic signals necessary for muscle repair and growth, thereby perpetuating the cycle of muscle wasting seen in sarcopenia. Addressing inflammation is therefore a key therapeutic target in mitigating the effects of sarcopenia and improving muscle health in the aging population.

Soft tissue manipulation (STM) is a noninvasive, nonpharmacological mechanotherapy employed by osteopathic physicians (referred to as “soft tissue OMT” [osteopathic manipulative treatment]), physiotherapists, and massage therapists, wherein soft tissues are subjected to mechanical forces delivered by hand or by an instrument [13]. Cells integrate those mechanical stimuli into mechanotransductive signaling pathways that regulate cellular behavior [13, 14]. Virtually all cells are mechanosensitive to their surrounding environment in that physical forces – eg, stretch, compression, etc – influence the physiology of tissues and ultimately the organism [13]. STM is utilized by practitioners to reduce inflammation, and it lowers levels of several pro-inflammatory mediators in human and rat soft tissue biopsies, in sera from rats, and in conditioned media from primary human dermal fibroblasts [15–23]. However, it is unknown if STM confers anti-inflammatory effects in sarcopenia.

In the present study, we utilized a rat model of sarcopenia due to disuse atrophy and examined the effects of STM on the recovery of muscle mass and regulation of pro-/anti-inflammatory cytokines. Atrophy was induced by suspending the animals by their tails for 2 weeks and was associated with increased markers of inflammation in skeletal muscle and serum. Following suspension, STM enhanced the effectiveness of re-ambulation (Re-A) on muscle mass recovery and was associated with changes in numerous cytokines in skeletal muscle and sera. Moreover, STM induced similar changes in muscle recovery in both treated and contralateral hindlimbs, raising the possibility that STM may exert systemic effects on the musculoskeletal system.

## Methods

### Animals

Ten-week-old male Brown Norway rats were purchased from Charles River Labs (Wilmington, MA) and randomly assigned to the following groups: weight-bearing control (Controls) maintained in standard cages, or one of three groups subjected to 2-week hindlimb suspension (HLS) in custom-modified cages. Tails were secured via foam tape to a pulley that allowed movement around the cage and access to food and water *ad lib*; food was provided via floor feeding while water was provided via a bottle affixed to the cage wall. Cages were changed after 1 week. Following suspension, some animals were immediately euthanized (HLS group), whereas others were transferred to standard cages and allowed to re-ambulate for 8 days +/− instrument-assisted soft tissue manipulation (IASTM, see below). Euthanasia was completed by asphyxiation and bilateral pneumothorax. Blood and tissue samples were obtained immediately postmortem as follows: blood was collected with a 16-gague needle through cardiac puncture; distal portions of the left and right quadriceps were collected and stored in −80 °C; left and right gastrocnemii (all collected by JWL only to ensure consistency in technique) were weighed and dried as described below. All animal procedures were performed in alignment with a protocol approved by the Indiana University Institutional Animal Care & Use Committee and national standards.

### Instrument-assisted soft tissue manipulation

A quantifiable soft tissue manipulation (QSTM) (Precision Care Technologies, Inc., Indianapolis, IN) handheld device (Q1-L) was utilized for manual delivery and standardization of localized force magnitude and stroke frequency application [24, 25]. The Q1-L treatment edge is similar to the GT3 device (Graston Technique, Indianapolis, IN) utilized for performing instrument-assisted soft tissue manipulation (IASTM). The treatment edge on the device, which is tooled to accommodate manipulation of small areas, was applied over four sessions beginning 1 day after release from HLS and then every other day thereafter. IASTM sessions were approximately 10 min in duration, with 5 min each to the upper and lower hindlimb, while the rats were under isoflurane-induced anesthesia (15.44063 ± 0.38 min average anesthesia per session). IASTM included applied curvilinear, longitudinal, and cross-fiber strokes at an average force of 2.72 N ± 0.11 N (i.e., 0.61 ± 0.02 lbs) at a frequency of 0.92 ± 0.12 Hz.

### Muscle weights

Ipsilateral (right) and contralateral (left) gastrocnemii were weighed utilizing an analytical balance at two time points: (1) immediately following collection; and (2) after 12 days of drying at 55 °C.

### Tissue homogenates

Quadriceps biopsies were homogenized in 1 X RIPA buffer (Cell Signaling, Danvers, MA) with 1 X Halt Protease and Phosphatase Inhibitor Cocktail (Thermo Fisher Scientific, Waltham, MA). After 10-min incubation on ice, samples were subjected to two 5-min cycles at full speed in a Bullet Blender (Next Advance, Troy, NY) then incubated for 30 min on ice. Samples were centrifuged at full speed for 15 min at 4 °C, and then the liquid phase was removed to a new tube. Protein concentration was determined utilizing a bicinchoninic acid (BCA) assay (Thermo Fisher Scientific, Waltham, MA) on a BioTek Synergy HTX plate reader (Agilent Technologies, Santa Clara, CA).

### Serum collection

Whole blood was collected immediately postmortem and placed on ice. Following centrifugation at 1 k × gravity for 7 min at 4 °C, the liquid phase was removed to a new tube and stored at −80 °C. Protein concentration was determined utilizing a BCA Assay (Thermo Fisher Scientific, Waltham, MA) on a BioTek Synergy HTX plate reader (Agilent Technologies, Santa Clara, CA).

### Cytokine arrays

A total of 500 μg total protein from ipsilateral quadriceps or serum was prepared utilizing equal amounts of protein for animals within a given group; specific numbers of animals are detailed in the respective figure or table legend. Pooled tissue homogenates and serum were analyzed utilizing the Proteome Profiler Rat XL Cytokine Array (R&D Systems, ARY030) as directed by the manufacturer with the following modification: the arrays were developed utilizing Western-Bright Sirius reagent (Advansta, San Jose, CA) on a C-Digit scanner (LI-COR, Lincoln, NE). Signal densities were determined utilizing Image Studio software package (LI-COR). For each membrane, signal density for a given analyte’s duplicate spots were expressed as relative to the average density of the reference spots on the same membrane. Relative densities were then normalized to the respective control condition as detailed in the text and/or figure legend.

### Statistical considerations

Statistical analyses were performed utilizing GraphPad Prism 10 as described in each respective figure legend or in the text. A p value of < 0.05 was considered significant.

### Funding

This study was supported by a grant from the American Osteopathic Association (AOA, Award Number 21085846, issued to JWL) and intramural funds from Marian University (issued to JWL).

## Results

### Hindlimb suspension results in skeletal muscle atrophy and changes in cytokine levels

**Figure 1:**
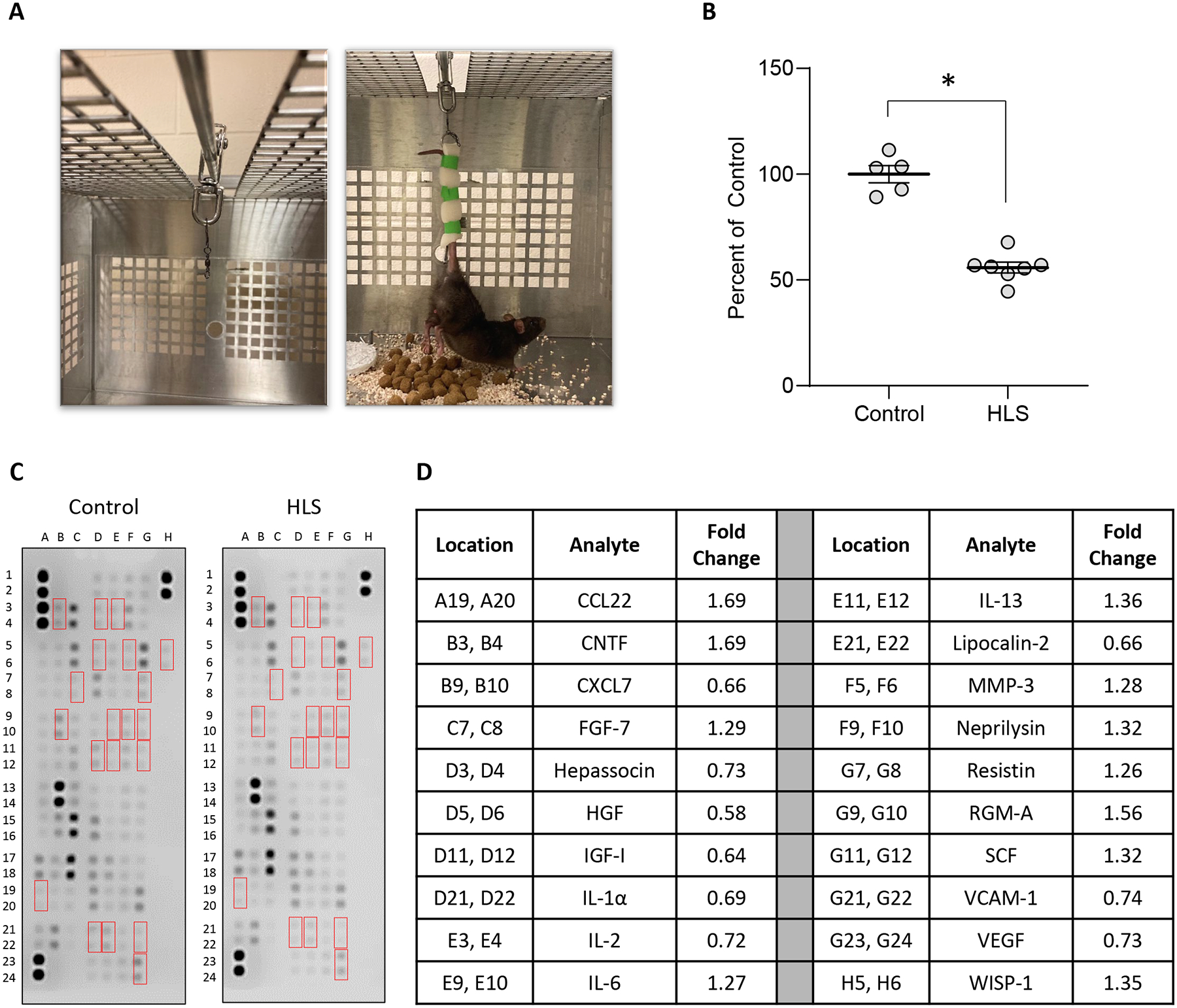
HLS results in skeletal muscle atrophy and changes in cytokine levels. (A) Images of HLS setup, wherein cages were modified with a bar and rolling pully (left) to which animal tails were affixed utilizing foam tape (right). (B) Mass of gastrocnemius immediately after sacrifice from weight-bearing controls (control) and animals subjected to 2-week HLS. Circles represent gastrocnemius mass from individual animals expressed as percent relative to the mean weight for the control group. Bar is mean ± standard error of the mean (SEM). n=5 for control and n=7 for HLS. Data were determined to be normally distributed by the Shapiro-Wilk test. * indicates p<0.05 by unpaired t-test. Raw weights obtained immediately after sacrifice and after 12 days of drying may be found in Supplemental Figures 1A and B. (C, D) membrane arrays utilizing quadricep homogenates obtained from control or HLS animals. Homogenates were pooled at equal ratios within groups for n=5 for control and n=6 for HLS. Red boxes in C indicate factors detailed in D, which are those altered by≥25 % in HLS compared to control. In D, data are expressed as fold change relative to control. Complete quantification may be found in Supplemental Table 1.

Rats were randomly assigned to the weight-bearing control group (Controls) or the HLS group, wherein animals were suspended by their tail to remove loading of body weight from their hindlimbs (Figure 1A). After a 2-week suspension, animals were euthanized and tissue samples were collected for determining the effects of unloading on skeletal muscle mass and expression levels of select cytokines in both skeletal muscle and serum. This revealed that 2 weeks of unloading is associated with an approximately 48 % decrease in wet weight of the gastrocnemius (Figure 1B); raw weights of gastrocnemii may be found in Supplemental Figure 1.

**Figure 2:**
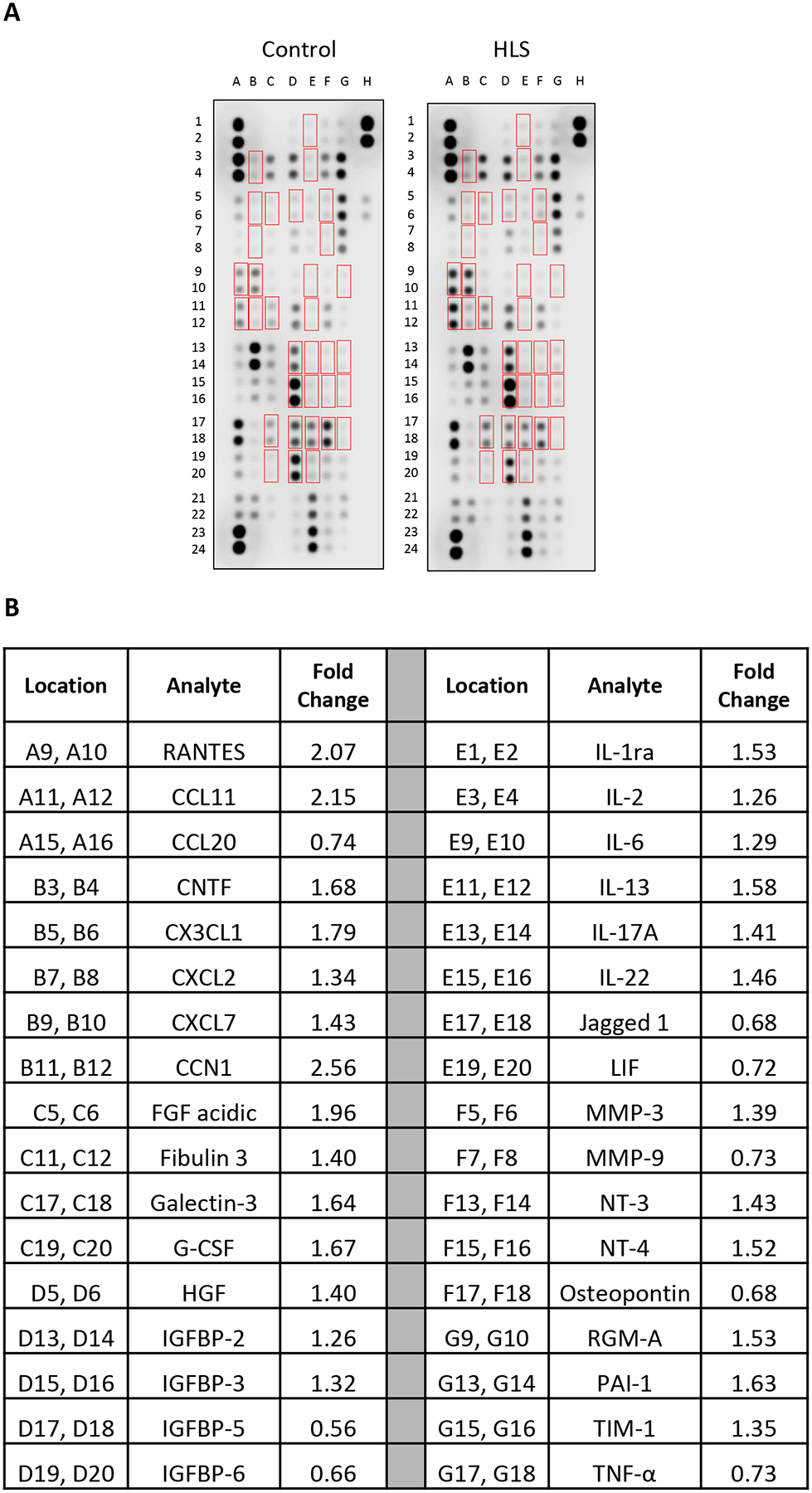
Membrane arrays utilizing sera obtained from control or HLS animals. Sera were pooled at equal ratios within groups for n=5 for control and n=7 for HLS. Red boxes in (A) indicate factors detailed in (B), which are those altered by≥25 % in HLS compared to control. (B) Data are expressed as fold change relative to control. Complete quantification may be found in Supplemental Table 2.

To examine the tissue-specific changes associated with HLS, homogenates from quadriceps biopsies were pooled within groups and subjected to membrane-based cytokine arrays that examine expression levels of nearly 80 targets simultaneously (Figure 1C and D). These assays detected modest changes in numerous cytokines with HLS (Supplemental Table 1), including 20 that were at least 25 % higher or lower compared to Controls (Figure 1C and D). Among these, the most strongly regulated cytokines were CTNF (1.69-fold increased), CCL22 (1.69-fold increased), and HGF (0.58-fold decreased). We next performed membrane arrays on serum pooled within groups to determine differences in circulating factors (Figure 2). This revealed HLS-associated changes in a greater number of serum factors than quadriceps, with levels of 34 targets at least 25 % higher or lower compared to Controls (Figure 2). Among these, the most strongly regulated cytokines were CCN1 (2.56-fold increased), CCL11 (2.15-fold increased), and FGF acidic (1.96-fold increased).

### STM enhances muscle mass recovery

**Figure 3:**
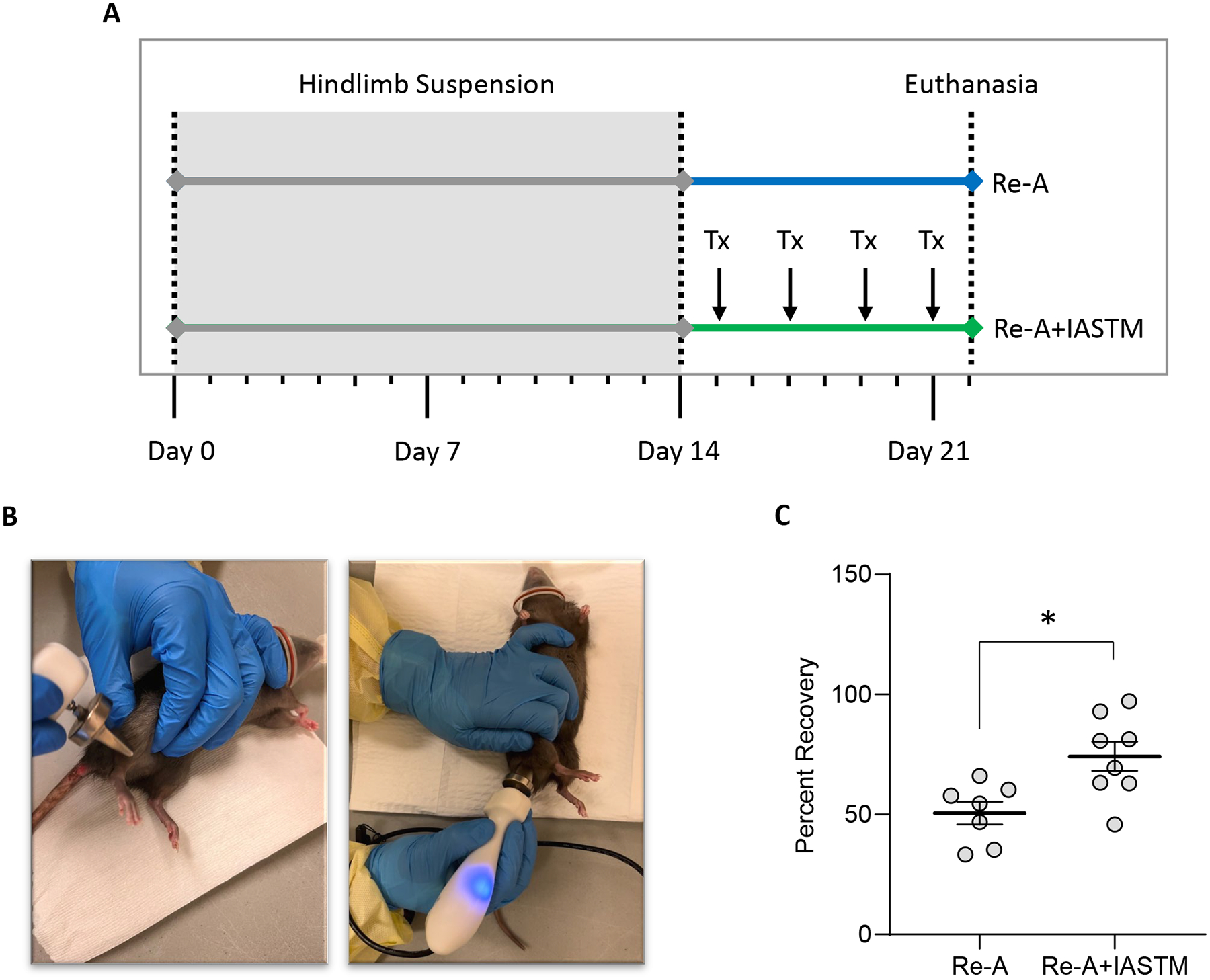
STM enhances muscle mass recovery following disuse atrophy. (A) Schematic of timeline for animals subjected to HLS then 8 days of Re-A or Re-A+IASTM. IASTM was carried out every other day beginning the day following release from HLS for a total of four sessions (Tx). All animals were euthanized 8 days following HLS. (B) Images of the IASTM technique. (C) Mass of ipsilateral gastrocnemius immediately after sacrifice from Re-A and Re-A+IASTM animals. Circles represent gastrocnemius mass from individual animals expressed as percent relative to the mean lost in the HLS group. Bar is mean ± standard error of the mean (SEM). n=7 for Re-A and n=8 for Re-A+IASTM. Data were determined to be normally distributed by the Shapiro-Wilk test. * indicates p<0.05 by unpaired t-test. Raw weights obtained immediately after sacrifice and after 12 days of drying may be found in Supplemental Figures 1A and B.

We next sought to examine the effects of STM on muscle mass recovery following disuse atrophy. After 2 weeks of tail suspension, rats were randomly assigned to re-ambulate or re-ambulate plus four sessions of IASTM (Re-A+IASTM) delivered every other day to one hindlimb (Figure 3A and B). Details of the IASTM may be found in the Methods Section; briefly, under isoflurane anesthesia (average 15.44 ± 0.38 min total), an IASTM device was applied in a stroking pattern to the upper and lower hindlimb for 5 min each at an average force of 2.72 N ± 0.11 N (i.e., 0.61 ± 0.02 lbs) at a frequency of 0.92 ± 0.12 Hz. After 8 days, animals in both groups were euthanized. This revealed that Re-A alone is associated with approximately 50 % recovery of gastrocnemius wet weight, whereas Re-A+IASTM is associated with approximately 74 % recovery (Figure 3C); raw weights of gastrocnemii may be found in Supplemental Figure 1.

### STM alters cytokine levels in skeletal muscle and serum

**Figure 4:**
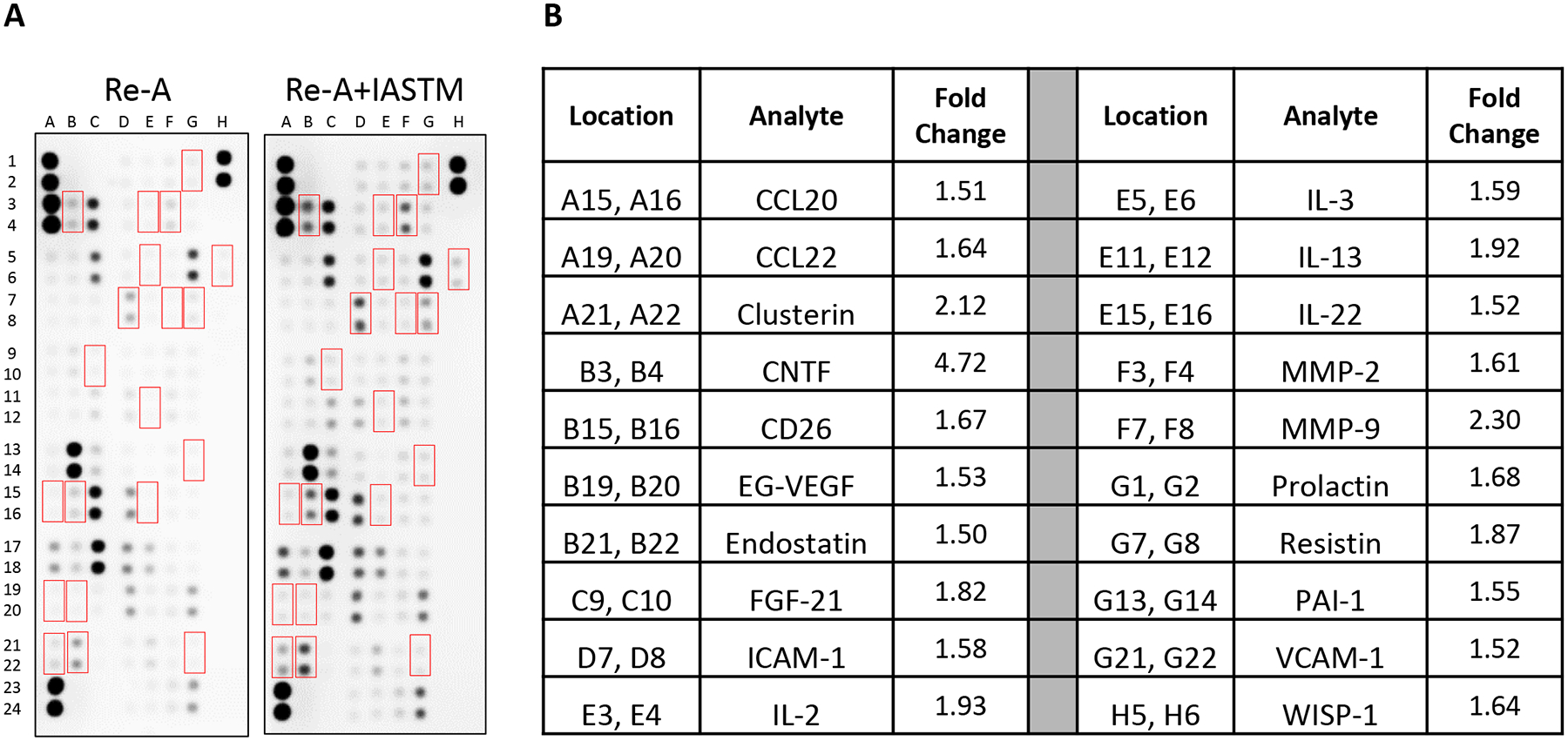
Membrane arrays utilizing quadricep homogenates obtained from animals subjected to HLS then 8 days of Re-A or Re-A+IASTM. Homogenates were pooled at equal ratios within groups for n=7 for Re-A and n=8 for Re-A+IASTM. Red boxes in (A) indicate factors detailed in B, which are those altered by≥25 % in HLS compared to control. (B) Data are expressed as fold change relative to control. Complete quantification may be found in Supplemental Table 3.

To examine the tissue-specific changes associated with IASTM, homogenates from treated (i.e., ipsilateral) quadriceps biopsies were pooled within groups and subjected to membrane-based cytokine arrays (Figure 4). These assays detected changes in numerous cytokines with IASTM (Supplemental Table 2), including 49 that were at least 25 % higher or lower compared to Re-A alone. The 20 most strongly regulated cytokines are detailed in Figure 4B, with CTNF (4.72-fold increased), MMP-9 (2.30-fold increased), and clusterin (2.12-fold increased) representing the three largest changes compared to the Re-A group.

**Figure 5:**
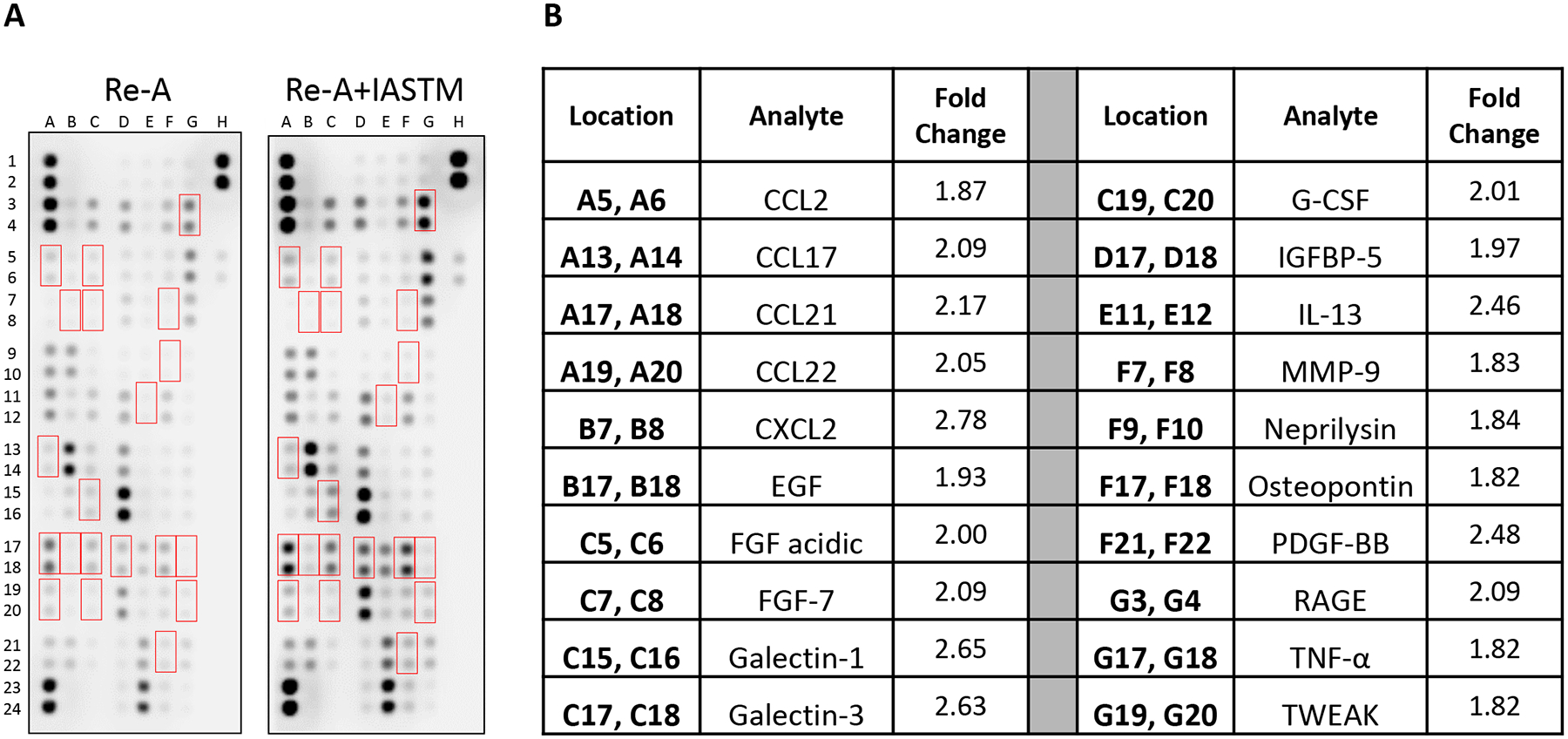
Membrane arrays utilizing sera homogenates obtained from animals subjected to HLS then 8 days of Re-A or Re-A+IASTM. Homogenates were pooled at equal ratios within groups for n=7 for Re-A and n=8 for Re-A+IASTM. Red boxes in (A) indicate factors detailed in (B), which are those altered by≥25 % in HLS compared to control. (B) Data are expressed as fold change relative to control. Complete quantification may be found in Supplemental Table 4.

We next utilized membrane arrays to determine the effects of STM on serum factors, which revealed changes in the levels of 53 targets at least 25 % higher or lower compared to Re-A alone (Figure 5). The 20 most strongly regulated cytokines are detailed in Figure 5B, with CXCL2 (2.78-fold increased), Galectin-1 (2.65-fold increased), and Galectin-3 (2.63-fold increased) representing the three largest changes compared to the Re-A group.

### Crossover effect of STM on muscle mass recovery

**Figure 6:**
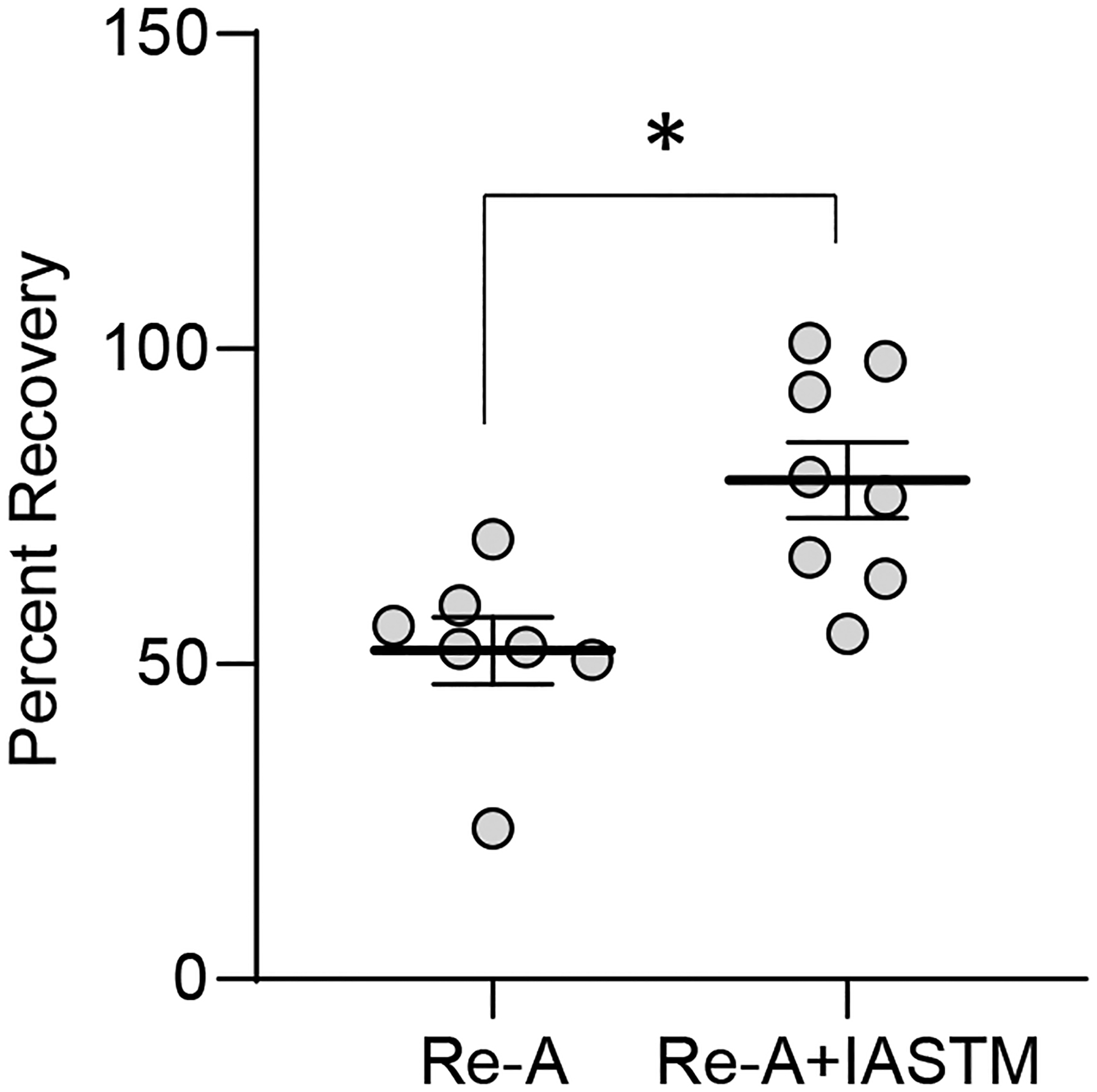
Mass of contralateral gastrocnemius immediately after sacrifice from Re-A and Re-A+IASTM animals. Circles represent gastrocnemius mass from individual animals expressed as percent relative to the mean lost in the HLS group. Bar is mean ± standard error of the mean (SEM). n=7 for Re-A and n=8 for Re-A+IASTM. Data were determined to be normally distributed by the Shapiro-Wilk test. * indicates p<0.05 by unpaired t-test. Raw weights obtained immediately after sacrifice and after 12 days of drying may be found in Supplemental Figures 1C and D.

Given the changes in systemic factors observed in rats subjected to IASTM, we examined the possibility for crossover effects on muscle mass recovery in contralateral hindlimbs. Analysis of gastrocnemius wet weight indicates that recovery of muscle mass is enhanced by IASTM in contralateral limbs, with Re-A alone associated with approximately 52 % recovery, whereas Re-A+IASTM was associated with approximately 79 % recovery (Figure 6); raw weights of contralateral gastrocnemii may be found in Supplemental Figure 1.

### Identification of putative therapeutic targets in disuse atrophy regulated by IASTM

Our analyses above were normalized in such a manner to aid in clear identification of factors altered by HLS compared to controls (Figures 1 and 2) or by IASTM compared to Re-A alone (Figures 4 and 5). However, this statistical method is not ideal to identify those factors that may play a role in the therapeutic benefit of IASTM on the recovery of muscle mass following disuse atrophy.

To aid in this identification, we normalized the membrane arrays to the weight-bearing condition and filtered for the following: Group 1) factors that were increased/decreased by HLS, regulated in the opposite direction by Re-A, and further regulated by Re-A+IASTM; Group 2) factors that were increased/decreased by HLS, unchanged by Re-A alone, but regulated by Re-A+IASTM in an opposite direction than HLS; Group 3) factors that were regulated in opposite directions by Re-A vs. Re-A+IASTM; Group 4) factors that were unchanged by HLS or Re-A but increased/decreased by Re-A+IASTM. A threshold of 25 % increase or decrease was utilized for all analyses.

Application of these criteria to homogenates from ipsilateral quadriceps may be found in Supplemental Table 3, and the 22 factors that meet any set of conditions are highlighted in Table 1. Similarly, application of these criteria to the full panel of serum factors may be found in Supplemental Table 4, and the 35 factors that meet any set of conditions are highlighted in Table 2.

## Discussion

In this study, we examined the ability of STM to enhance recovery of muscle mass following disuse atrophy. We found greater grains in ipsilateral muscle mass among animals receiving IASTM, along with changes in the levels of numerous cytokines in both treated tissue and in serum. These findings support the notion that STM may be a useful therapy for promoting musculoskeletal function in conditions such as sarcopenia. In particular, our molecular characterization of changes related to IASTM add further evidence that this force-based manipulation exerts anti-inflammatory effects in both target tissues and systemically. This is important because chronic inflammation is an important factor in numerous conditions and STM may be a noninvasive, nonpharmacological means of therapy [26].

Our study identifies numerous cytokines whose expression levels in skeletal muscle and/or serum are altered by hindlimb unloading and Re-A. To the best of our knowledge, our arrays provide the first information on many of these in the hindlimb-unloaded model, whereas for others, our findings corroborate prior reports. For instance, we and others have found that levels of ciliary neurotrophic factor (CNTF) and insulin-like growth factor binding protein 5 (IGFBP5) increase in skeletal muscle with unloading [27, 28]. Our results are also consistent with a report that Re-A following unloading is associated with increased IGFBP5 levels in muscle [29]. Likewise, our results are similar to others demonstrating increased levels of the pro-inflammatory cytokine interleukin (IL)-6 in circulation with hindlimb unloading [30]. That said, there are numerous factors and pathways reported by others to be regulated by hindlimb unloading and/or Re-A (such as anabolic and apoptotic mechanisms) that were not examined in our study [31].

Taken together, it is clear that hindlimb unloading is associated with robust and widespread changes in molecular physiology that lead to muscular atrophy. Thus, it is striking that STM enhances the ability of Re-A to regain muscle mass lost due to HLS. In this regard, our study utilizing human-delivered IASTM with standardized force application is consistent with another report, wherein STM was applied via a computer-controlled robotic arm [32]. In that study, cyclic compressive loading was delivered at similar timepoints as our study (i.e., every other day for a period of 8 days following 2-week HLS) but delivered to a single muscle (gastrocnemius) in older animals (10 months of age) at a higher force (4.5 N), lower frequency (0.5 Hz), and for a longer time (30 min) [32]. Despite these differences, both Miller et al. [32] and we report enhanced muscle recovery in STM-treated sites. Whereas Miller et al. [32] provided robust information on the effects of STM to increase protein synthesis and activate anabolic pathways, our findings detail numerous cytokines regulated by STM in both the target tissue and serum. Notably, several factors we observe to be regulated by mechanical stimulation were also shown to be regulated by STM in healthy humans, in a rat model of low back pain, as well as in a mouse model of stroke – including CNTF, IL-1β, IL-2, IL-3, IL-13, intercellular adhesion molecule (ICAM)-1, and tumor necrosis factor (TNF)-α [22, 33, 34].

Overall, these studies provide strong evidence that STM is a force-based manipulation that exerts widespread molecular-level effects in target tissues. It is important to note, however, that both our study and Miller et al. [32] report a crossover effect of STM. Similarly, a separate study in which STM was delivered during the HLS phase (rather than afterward) is associated with increased myofibrillar protein synthesis and decreased protein degradation in both treated and nontreated limbs [35]. These findings are reminiscent of the crossover effects reported in several other studies examining STM or resistance training with or without additional electrical stimulation [36–42]. Several of these studies argued for a neurogenic origin, but it is presently unclear what mechanism(s) underlie this phenomenon. It is worth noting, however, that several studies (including this one) report STM-induced changes in circulating factors, raising the possibility that endocrine-like effects may participate in nonlocal effects [15, 16, 22, 23, 34, 43]. These reports are supported by *in vitro* studies in which isolated cells subjected to a mechanical force simulating STM alter the production of numerous secreted factors including pro-/anti-inflammatory cytokines [17–21].

Our work and the previously referenced studies detail recent advancements in the understanding of therapeutic STM and elucidating the associated molecular-level effects. This provides a foundation for future work to examine STM in conditions of musculoskeletal atrophy including age-related sarcopenia, prolonged bedrest or immobility, and spaceflight. However, there are several important limitations to the present study. First, it remains to be determined which of these effects are directly attributable to the force itself vs. those influenced by additional behavioral and/or psychosocial inputs. For instance, our study cannot comment on the possibility that animals receiving STM are more active compared to Re-A controls, which could contribute to the enhanced muscle recovery in treated and nontreated limbs. Consistent with the necessity of physical activity, there are several reports that STM delivered during HLS is not associated with a therapeutic benefit [35, 44, 45]. Additionally, we cannot definitively comment on which protein(s) regulated by IASTM are most important to the recovery of muscle mass. We are also aware that the therapeutic benefit of STM may diminish with age and that findings from our young adult animals may not be representative of the most likely application in humans [35, 44, 46]. Other limitations include our relatively small sample size, inclusion a single sex, analysis of only a single timepoint, lack of long-term follow-up examination, and absence of muscle function characterization. These are primarily due to the challenges associated with this experimental system, which is expensive, logistically complex, staff-intensive, and time-consuming. Moreover, the HLS model exerts considerable stress on the animals and requires extraordinary involvement from veterinary staff to ensure humane animal care.

## Conclusions

Our study adds further support for the role of manual therapy in musculoskeletal health. In particular, STM may hold promise for recovering muscle mass and function related in conditions of disuse atrophy. Our findings suggest that STM orchestrates a complex interaction of anti-inflammatory and pro-inflammatory factors and an immune response that is beneficial in the recovery of muscle loss superior to physical activity alone. Despite some limitations, our results are strikingly consistent with several other studies and provide a strong foundation for future research. We encourage investigators to design clinical trials examining the impact of STM on the tissue-level and circulating factors highlighted in our study. Such translation of our work into clinical studies would provide solid evidence for this noninvasive treatment option for managing sarcopenia, ultimately contributing to better patient outcomes and advancing the fields of manual therapy.

## Supplementary Material

Supplemental Table 6

Supplemental Table 5

Supplemental Table 4

Supplemental Table 2

Supplemental Table 1

Supplemental Figure

Supplemental Table 3

## Figures and Tables

**Table 1:** Comparison of cytokine levels in tissue homogenates of quadriceps from weight-bearing controls (Control) or animals subjected to hindlimb suspension (HLS) then immediately sacrificed, permitted 8 days of re-ambulation (Re-A), or re-ambulation plus instrument-assisted soft tissue manipulation (Re-A+IASTM). Total protein was pooled at equal ratios for n=5 for Control, n=6 for HLS, n=7 for Re-A, and n=8 for Re-A+IASTM animals. Data are mean signal density relative to the average reference spot density normalized to Control. Green and pink highlighting indicates≥25 % increase or decrease, respectively, compared to Control.

	Analyte	Control	HLS	Re-A	Re-A+IASTM
A15, A16	CCL20	1	0.90	0.63	0.90
B15, B16	DPPIV	1	0.87	0.75	1.24
C9, C10	FGF-21	1	1.07	0.72	1.30
C19, C20	G-CSF	1	1.07	1.10	1.38
D1, D2	GM-CSF	1	0.77	0.74	1.02
D3, D4	Hepassocin	1	0.73	0.95	1.31
E3, E4	IL-2	1	0.73	0.66	1.25
E5, E6	IL-3	1	0.76	0.70	1.11
E7, E8	IL-4	1	0.95	0.95	1.35
E15, E16	IL-22	1	0.99	1.18	1.78
F3, F4	MMP-2	1	1.01	0.92	1.48
F7, F8	MMP-9	1	1.24	0.85	1.94
F11, F12	N0V	1	1.01	1 .08	1.37
F13, F14	NT-3	1	1.03	1 .07	1.38
F15, F16	NT-4	1	0.85	0.92	1.34
G1, G2	Prolactin	1	0.80	0.92	1.54
G3, G4	RAGE	1	1.00	0.87	1.28
G5, G6	RBP4	1	1.04	1.02	1.38
G13, G14	PAI-1	1	1.19	1.01	1.56
G15, G16	TIM-1	1	1.06	0.97	1.41
G17, G18	TNF-α	1	0.90	1.03	1.53
G21, G22	VCAM-1	1	0.74	0.92	1.37

**Table 2:** Comparison of cytokine levels in sera from weight-bearing controls (Control) or animals subjected to hindlimb suspension (HLS) then immediately sacrificed, permitted 8 days of re-ambulation (Re-A), or re-ambulation plus instrument-assisted soft tissue manipulation (Re-A+IASTM). Total protein was pooled at equal ratios for n=5 for Control, n=7 for HLS, n=7 for Re-A, and n=8 Re-A+IASTM animals. Data are mean signal density relative to the average reference spot density normalized to Control. Green and pink highlighting indicates≥25 % increase or decrease, respectively, compared to Control.

	Analyte	Control	HLS	Re-A	Re-A+IASTM
A5, A6	CCL2	1	1.05	0.68	1.27
A13, A14	CCL17	1	0.92	0.54	1.14
A15, A16	CCL20	1	0.74	0.63	0.87
A17, A18	CCL21	1	1.03	0.41	0.90
A19, A20	CCL22	1	0.92	0.41	0.84
B3, B4	CNTF	1	1.68	0.75	0.99
B5, B6	CX3CL1	1	1.79	0.70	1.00
B9, B10	CXCL7	1	1.43	0.73	0.98
B13, B14	Cystatin C	1	0.90	0.66	0.92
B15, B16	DPPIV	1	0.85	0.62	0.89
B19, B20	EG-VEGF	1	1.07	0.59	0.96
C11, C12	Fibulin 3	1	1.40	0.63	0.92
C13, C14	Flt-3 Ligand	1	0.85	0.70	0.91
C15, C16	Galectin-1	1	1.21	0.94	2.49
C17, C18	Galectin-3	1	1.64	0.71	1.87
C19, C20	G-CSF	1	1.66	0.61	1.19
D7, D8	ICAM-1	1	1.19	0.57	0.92
D9, D10	IFN-γ	1	0.83	0.52	0.78
D17, D18	IGFBP-5	1	0.56	0.56	1.10
D19, D20	IGFBP-6	1	0.66	0.65	1.11
E11, E12	IL-13	1	1.58	0.70	1.82
E17, E18	Jagged 1	1	0.68	0.64	1.09
E19, E20	LIF	1	0.72	0.55	0.82
F5, F6	MMP-3	1	1.39	0.70	0.87
F7, F8	MMP-9	1	0.73	0.51	0.93
F9, F10	Neprilysin	1	0.87	0.53	0.96
F17, F18	Osteopontin	1	0.68	0.63	1.14
F19, F20	Osteoprotegerin	1	0.88	0.68	1.15
F21, F22	PDGF-BB	1	1.24	0.79	1.95
G1, G2	Prolactin	1	0.87	0.91	0.50
G3, G4	RAGE	1	0.86	0.56	1.16
G9, G10	RGM-A	1	1.53	0.71	1.15
G11, G12	SCF	1	1.17	0.67	1.20
G17, G18	TNF-α	1	0.73	0.59	1.06
G19, G20	TWEAK	1	0.81	0.67	1.23

## Data Availability

The datasets generated and/or analyzed during the current study are available from the corresponding author on reasonable request.
