# Supplementary material for: Soft tissue manipulation enhances recovery of muscle mass in a disuse model of sarcopenia
Source: J Osteopath Med. Author manuscript; Available in PMC 2025 Aug 16. (PMC12353430; doi:10.1515/jom-2024-0247)
Supplement: Supplemental Table 5 [file NIHMS2078058-supplement-Supplemental_Table_5.pdf]

Supplemental Table 5. Comparison of cytokine levels in in tissue homogenates of quadriceps from weight-bearing controls (Control) or animals subjected to hindlimb suspension then immediately sacrificed (HLS), permitted eight days of re-ambulation (Re-A), or re-ambulation plus instrument-assisted soft tissue manipulation (Re-A+IASTM). Total protein was pooled at equal ratios for n=5 for Control, n=6 for HLS, n=7 Re-A and n=8 Re-A+IASTM animals. Data are mean signal density relative to the average reference spot density normalized to Control. Green and pink highlight indicates ≥25% increase or decrease, respectively, compared to Control.

	Analyte	Control	HLS	Re-A	Re-A+IASTM
**A3, A4**	Adiponectin/Acrp30	1	0.96	1.00	0.96
**A5, A6**	CCL2/JE/MCP-1	1	1.18	1.53	1.76
**A7, A8**	CCL3/CCL4/MIP-1α/β	1	1.11	1.05	1.46
**A9, A10**	CCL5/RANTES	1	1.00	1.19	1.34
**A11, A12**	CCL11/Eotaxin	1	0.98	0.99	1.30
**A13, A14**	CCL17/TARC	1	1.05	0.80	0.92
**A15, A16**	CCL20/MIP-3α	1	0.90	0.63	0.90
**A17, A18**	CCL21/6Ckine	1	1.19	0.90	1.20
**A19, A20**	CCL22/MDC	1	1.69	1.23	2.02
**A21, A22**	Clusterin	1	1.04	0.85	1.73
**B3, B4**	CNTF	1	1.69	1.02	2.80
**B5, B6**	CX3CL1/Fractalkine	1	1.25	1.30	1.62
**B7, B8**	CXCL2/GROβ/MIP-2/CINC-3	1	0.97	1.22	1.55
**B9, B10**	CXCL7/Thymus Chemokine-1	1	0.66	0.53	0.71
**B11, B12**	Cyr61/CCN1	1	1.11	1.26	1.51
**B13, B14**	Cystatin C	1	1.13	0.90	0.83
**B15, B16**	DPPIV/CD26	1	0.87	0.75	1.24
**B17, B18**	EGF	1	0.90	1.07	0.89
**B19, B20**	EG-VEGF/PK1	1	1.24	1.17	1.79
**B21, B22**	Endostatin	1	0.85	0.79	1.19
**C3, C4**	Fetuin A/AHSG	1	0.91	0.85	1.07
**C5, C6**	FGF acidic	1	1.06	0.96	1.17
**C7, C8**	FGF-7/KGF	1	1.29	0.94	1.11
**C9, C10**	FGF-21	1	1.07	0.72	1.30
**C11, C12**	Fibulin 3	1	1.11	0.86	1.24
**C13, C14**	Flt-3 Ligand	1	1.07	1.02	1.02
**C15, C16**	Galectin-1	1	1.06	0.91	0.88
**C17, C18**	Galectin-3	1	1.25	1.02	1.16
**C19, C20**	G-CSF	1	1.07	1.10	1.38
**C21, C22**	GDF-15	1	0.87	0.91	1.03
**D1, D2**	GM-CSF	1	0.77	0.74	1.02
**D3, D4**	Hepassocin	1	0.73	0.95	1.31
**D5, D6**	HGF	1	0.59	0.81	1.06
**D7, D8**	ICAM-1/CD54	1	0.76	1.06	1.67
**D9, D10**	IFN-γ	1	1.18	0.89	1.29
**D11, D12**	IGF-I	1	0.64	1.24	1.47
**D13, D14**	IGFBP-2	1	1.18	1.22	1.74
**D15, D16**	IGFBP-3	1	0.85	1.14	1.43
**D17, D18**	IGFBP-5	1	1.17	1.36	1.40
**D19, D20**	IGFBP-6	1	1.19	1.22	1.49
**D21, D22**	IL-1α/IL-1F1	1	0.69	0.93	0.91
**D23, D24**	IL-1β/IL-1F2	1	1.03	1.63	1.50
**E1, E2**	IL-1ra/IL-1F3	1	0.99	0.85	1.18
**E3, E4**	IL-2	1	0.73	0.66	1.25
**E5, E6**	IL-3	1	0.76	0.70	1.11
**E7, E8**	IL-4	1	0.95	0.95	1.35
**E9, E10**	IL-6	1	1.27	0.96	1.34
**E11, E12**	IL-13	1	1.36	0.99	1.89
**E13, E14**	IL-17A	1	1.15	1.15	1.22

**E15, E16**	IL-22	1	0.99	1.18	1.78
**E17, E18**	Jagged 1	1	1.14	1.23	1.50
**E19, E20**	LIF	1	0.93	1.00	1.17
**E21, E22**	Lipocalin-2/NGAL	1	0.66	1.08	1.28
**E23, E24**	LIX	1	1.12	1.25	1.45
**F1, F2**	MAG/Siglec-4a	1	1.03	0.84	1.22
**F3, F4**	MMP-2	1	1.01	0.92	1.48
**F5, F6**	MMP-3	1	1.28	1.16	1.72
**F7, F8**	MMP-9	1	1.24	0.85	1.94
**F9, F10**	Neprilysin/CD10	1	1.32	1.00	1.35
**F11, F12**	NOV/CCN3	1	1.01	1.08	1.37
**F13, F14**	NT-3	1	1.03	1.07	1.38
**F15, F16**	NT-4	1	0.85	0.92	1.34
**F17, F18**	Osteopontin (OPN)	1	1.00	0.77	1.04
**F19, F20**	Osteoprotegerin/TNFRSF11B	1	1.02	1.03	0.85
**F21, F22**	PDGF-BB	1	0.76	1.02	0.98
**F23, F24**	Pref-1/DLK1/FA1	1	1.09	1.51	1.69
**G1, G2**	Prolactin	1	0.80	0.92	1.54
**G3, G4**	RAGE	1	1.00	0.87	1.28
**G5, G6**	RBP4	1	1.04	1.02	1.38
**G7, G8**	Resistin	1	1.26	1.08	2.02
**G9, G10**	RGM-A	1	1.56	1.27	1.57
**G11, G12**	SCF	1	1.32	1.11	1.48
**G13, G14**	Serpin E1/PAI-1	1	1.19	1.01	1.56
**G15, G16**	TIM-1/KIM-1/HAVCR	1	1.06	0.97	1.41
**G17, G18**	TNF-α	1	0.90	1.03	1.53
**G19, G20**	TWEAK/TNFSF12	1	0.98	1.01	1.08
**G21, G22**	VCAM-1/CD106	1	0.74	0.92	1.37
**G23, G24**	VEGF	1	0.73	1.34	1.35
**H5, H6**	WISP-1/CCN4	1	1.35	1.26	2.08
