# Supplementary material for: Soft tissue manipulation enhances recovery of muscle mass in a disuse model of sarcopenia
Source: J Osteopath Med. Author manuscript; Available in PMC 2025 Aug 16. (PMC12353430; doi:10.1515/jom-2024-0247)
Supplement: Supplemental Table 2 [file NIHMS2078058-supplement-Supplemental_Table_2.pdf]

Supplemental Table 2. Comparison of cytokine levels in sera from weight-bearing controls (Control) versus hindlimb suspension (HLS) groups. Total protein was pooled at equal ratios for n=5 Control and n=7 HLS animals. Data are mean signal density relative to the average reference spot density normalized to Control. Green and pink highlight indicates ≥25% increase or decrease, respectively, compared to Controls.

	Analyte	Control	HLS
**A3, A4**	Adiponectin/Acrp30	1	1.15
**A5, A6**	CCL2/JE/MCP-1	1	1.05
**A7, A8**	CCL3/CCL4/MIP-1α/β	1	1.01
**A9, A10**	CCL5/RANTES	1	2.07
**A11, A12**	CCL11/Eotaxin	1	2.15
**A13, A14**	CCL17/TARC	1	0.92
**A15, A16**	CCL20/MIP-3α	1	0.74
**A17, A18**	CCL21/6Ckine	1	1.03
**A19, A20**	CCL22/MDC	1	0.92
**A21, A22**	Clusterin	1	0.93
**B3, B4**	CNTF	1	1.68
**B5, B6**	CX3CL1/Fractalkine	1	1.79
**B7, B8**	CXCL2/GROβ/MIP-2/CINC-3	1	1.34
**B9, B10**	CXCL7/Thymus Chemokine-1	1	1.43
**B11, B12**	Cyr61/CCN1	1	2.56
**B13, B14**	Cystatin C	1	0.90
**B15, B16**	DPPIV/CD26	1	0.85
**B17, B18**	EGF	1	1.09
**B19, B20**	EG-VEGF/PK1	1	1.07
**B21, B22**	Endostatin	1	0.88
**C3, C4**	Fetuin A/AHSG	1	1.24
**C5, C6**	FGF acidic	1	1.96
**C7, C8**	FGF-7/KGF	1	0.94
**C9, C10**	FGF-21	1	1.24
**C11, C12**	Fibulin 3	1	1.40
**C13, C14**	Flt-3 Ligand	1	0.85
**C15, C16**	Galectin-1	1	1.21
**C17, C18**	Galectin-3	1	1.64
**C19, C20**	G-CSF	1	1.66
**C21, C22**	GDF-15	1	0.95
**D1, D2**	GM-CSF	1	0.95
**D3, D4**	Hepassocin	1	1.04
**D5, D6**	HGF	1	1.40
**D7, D8**	ICAM-1/CD54	1	1.19
**D9, D10**	IFN-γ	1	0.83
**D11, D12**	IGF-I	1	1.08
**D13, D14**	IGFBP-2	1	1.26

**D15, D16**	IGFBP-3	1	1.32
**D17, D18**	IGFBP-5	1	0.56
**D19, D20**	IGFBP-6	1	0.66
**D21, D22**	IL-1α/IL-1F1	1	1.13
**D23, D24**	IL-1β/IL-1F2	1	1.12
**E1, E2**	IL-1ra/IL-1F3	1	1.53
**E3, E4**	IL-2	1	1.26
**E5, E6**	IL-3	1	1.01
**E7, E8**	IL-4	1	1.10
**E9, E10**	IL-6	1	1.29
**E11, E12**	IL-13	1	1.58
**E13, E14**	IL-17A	1	1.41
**E15, E16**	IL-22	1	1.46
**E17, E18**	Jagged 1	1	0.68
**E19, E20**	LIF	1	0.72
**E21, E22**	Lipocalin-2/NGAL	1	0.87
**E23, E24**	LIX	1	1.21
**F1, F2**	MAG/Siglec-4a	1	0.77
**F3, F4**	MMP-2	1	1.04
**F5, F6**	MMP-3	1	1.39
**F7, F8**	MMP-9	1	0.73
**F9, F10**	Neprilysin/CD10	1	0.87
**F11, F12**	NOV/CCN3	1	1.07
**F13, F14**	NT-3	1	1.43
**F15, F16**	NT-4	1	1.52
**F17, F18**	Osteopontin (OPN)	1	0.68
**F19, F20**	Osteoprotegerin/TNFRSF11B	1	0.88
**F21, F22**	PDGF-BB	1	1.24
**F23, F24**	Pref-1/DLK1/FA1	1	0.92
**G1, G2**	Prolactin	1	0.87
**G3, G4**	RAGE	1	0.86
**G5, G6**	RBP4	1	1.04
**G7, G8**	Resistin	1	0.92
**G9, G10**	RGM-A	1	1.53
**G11, G12**	SCF	1	1.17
**G13, G14**	Serpin E1/PAI-1	1	1.63
**G15, G16**	TIM-1/KIM-1/HAVCR	1	1.35
**G17, G18**	TNF-α	1	0.73
**G19, G20**	TWEAK/TNFSF12	1	0.81
**G21, G22**	VCAM-1/CD106	1	0.94
**G23, G24**	VEGF	1	0.87
**H5, H6**	WISP-1/CCN4	1	0.76
