# Supplementary figures and images for: Soft tissue manipulation enhances recovery of muscle mass in a disuse model of sarcopenia
Source: J Osteopath Med. Author manuscript; Available in PMC 2025 Aug 16. (PMC12353430; doi:10.1515/jom-2024-0247)

**A**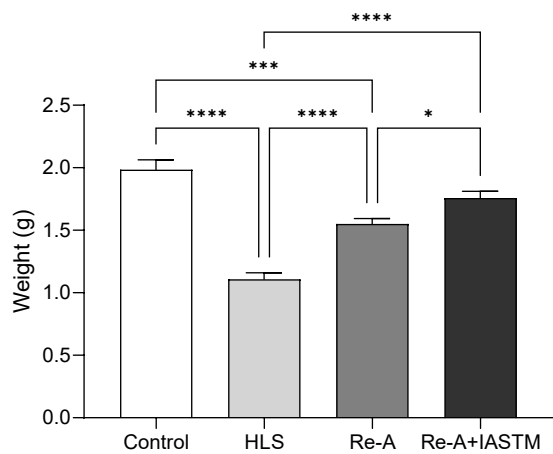**B**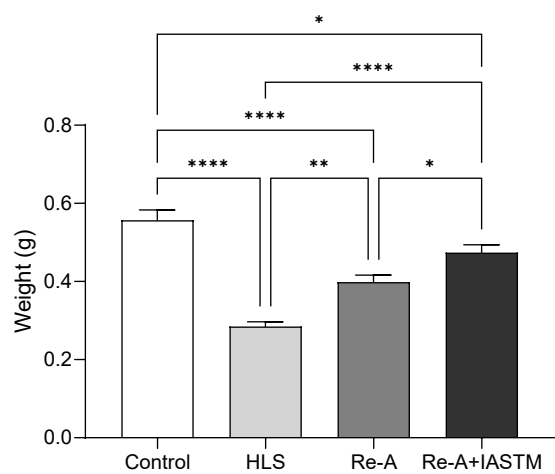**C**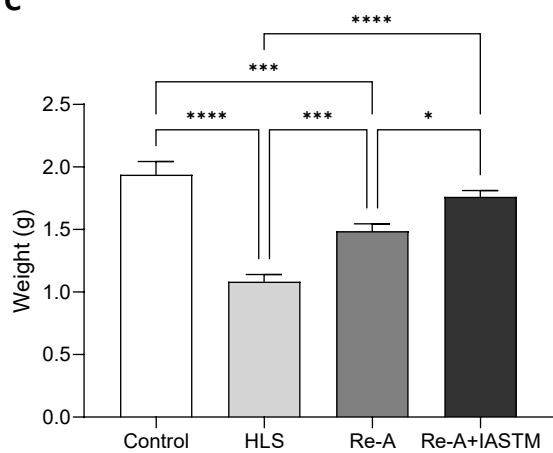**D**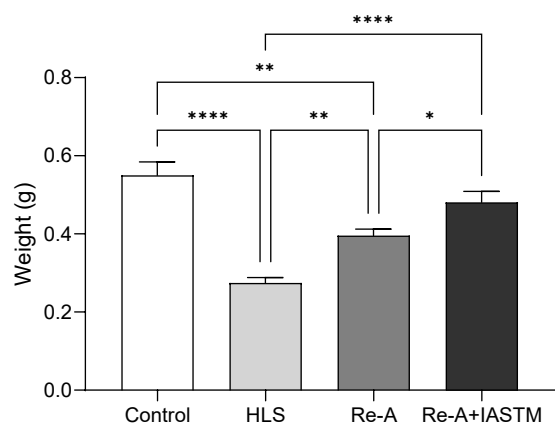

Supplement: Supplemental Figure [file NIHMS2078058-supplement-Supplemental_Figure.pdf]
